# Time-Shared Twin Memristor Crossbar Reducing the Number of Arrays by Half for Pattern Recognition

**DOI:** 10.1186/s11671-017-1973-4

**Published:** 2017-03-21

**Authors:** Son Ngoc Truong, Khoa Van Pham, Wonsun Yang, Anjae Jo, Mi Jung Lee, Hyun-Sun Mo, Kyeong-Sik Min

**Affiliations:** 1 School of Electrical Engineering, Kookmin University, Seoul, Korea; 2 School of Advanced Materials Engineering, Kookmin University, Seoul, Korea

**Keywords:** Time-shared twin memristor crossbar, Twin memristor crossbar, Pattern recognition

## Abstract

In this paper, we propose a new time-shared twin memristor crossbar for pattern-recognition applications. By sharing two memristor arrays at different time, the number of memristor arrays can be reduced by half, saving the crossbar area by half, too. To implement the time-shared twin memristor crossbar, we also propose CMOS time-shared subtractor circuit, in this paper. The operation of the time-shared twin memristor crossbar is verified using 3 × 3 memristor array which is made of aluminum film and carbon fiber. Here, the crossbar array is programmed to store three different patterns. When we apply three different input vectors to the array, we can verify that the input vectors are well recognized by the proposed crossbar. Moreover, the proposed crossbar is tested for the recognition of complicated gray-scale images. Here, 10 images with 32 × 32 pixels are applied to the proposed crossbar. The simulation result verifies that the input images are recognized well by the proposed crossbar, even though the noise level of each image is varied from −10 to +10 dB.

## Background

Memristor crossbars have been studied for many years for neuromorphic pattern recognitions [1–5]. Memristor crossbars can be thought very suitable to pattern recognition, in which all the columns of crossbar can be compared with the input pattern to find the best match simultaneously. Once the best-matched column is decided, the rest of columns are inhibited according to winner-take-all algorithm [3, 4].

Memristors which are used in pattern recognition can be either analog or binary. If we use analog memristor which can change its memristance gradually, pattern matching can be more accurate and demand a smaller number of memristors in crossbar array [6, 7]. However, analog memristor is more difficult to fabricate and more susceptible to noise and statistical variation than binary memristor [3]. Moreover, the number of memristive materials that show analog behavior is much smaller than the number of binary memristors. Based on these facts, binary memristors are used in pattern-matching crossbar, in this paper.

For the pattern-matching crossbar, we already proposed twin memristor crossbar (TMC) which could replace complementary memristor crossbar (CMC) [4]. CMC uses two memristor arrays of M^+^ and M^−^ to perform the exclusive NOR (XNOR) operation, where the M^+^ and M^−^ arrays are applied by the input vector and the inversion, respectively [3]. One thing to note here is that the number of low-resistance state (LRS) is very important in terms of sneak-path leakage because the leakage current flows mainly through LRS rather than high resistance state (HRS). In CMC, the total number of LRS in M^+^ and M^−^ arrays cannot be reduced at all, even though we use image compression algorithms such as discrete cosine transform (DCT) [4]. In CMC, M^+^ and M^−^ arrays are complementary to each other [3, 4]. It means that the same number of LRS in M^−^ array is increased always, though we reduce the number of LRS in M^+^ array using DCT [4]. Thus, the image compression becomes meaningless in CMC.

Unlike CMC, TMC uses two identical M^+^ arrays for performing XNOR operation. It means that the total number of LRS in the two identical arrays can be significantly reduced by using DCT, as explained well in the previous publication [4]. Based on TMC, we propose to apply a new time-sharing concept to TMC for reducing the number of TMC arrays by half, in this paper.

## Method

**Fig. 1:**
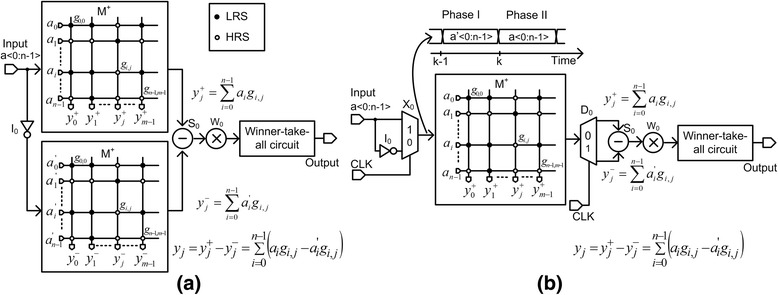
The conceptual diagram of twin memristor crossbars (TMCs) for pattern recognition. **a** The previous twin memristor crossbar (TMC) with two identical M^+^ arrays [4] and **b** the proposed time-shared TMC, where the number of crossbar arrays is reduced by half

Figure 1a shows the previous TMC with two identical M^+^ arrays. Here, the time-sharing concept is not used in Fig. 1a. The XNOR operation in TMC is expressed by the following Eq. (1) [4].

1 $$ {y}_j={y}_j^{+}-{y}_j^{-}={\displaystyle \sum_{i=0}^{n-1}\left({a}_i{g}_{i, j}-{a}_i^{\hbox{'}}{g}_{i, j}\right)} $$

Using Eq. (1), we can measure the amount of similarity between the input vector and the stored pattern in TMC arrays. Here, the input vector is represented by *a*_0_, *a*_1_, …, *a*_*n* − 1_ which enters the upper M^+^ array. *a* ^'^_0_, *a* ^'^_1_, …, *a* ^'^_*n* − 1_ are the inversion of the input vector *a*_0_, *a*_1_, …, *a*_*n* − 1_ which enters the lower M^+^ array in Fig. 1a. The pattern stored at column j is represented by *g*_0,*j*_, *g*_1,*j*_, …, *g*_*n* − 1,*j*_. I_0_ is the inverter in Fig. 1a. S_0_ and W_0_ are the subtractor and weighting circuit, respectively, in Fig. 1a. S_0_ and W_0_ can be designed using CMOS current mirror very easily [4]. ${y}_j^{+}$ and ${y}_j^{-}$ can be obtained from the jth column currents of the upper M^+^ and lower M^+^ arrays, respectively, in Fig. 1a. *y*_*j*_ means the amount of similarity of jth column with the input vector. Here, we assume that two jth columns in the upper and lower M^+^ arrays can store the same image in Fig. 1a. The number of columns in M^+^ array is as many as “m,” as shown in Fig. 1a. If we compare *y*_*j*_ values from j = 0 to m−1, we can know the largest *y*_*j*_ means the best matched column with the input vector. The largest *y*_*j*_ can be chosen by the winner-take-all circuit, as shown in Fig. 1a [3, 4].

**Fig. 2:**
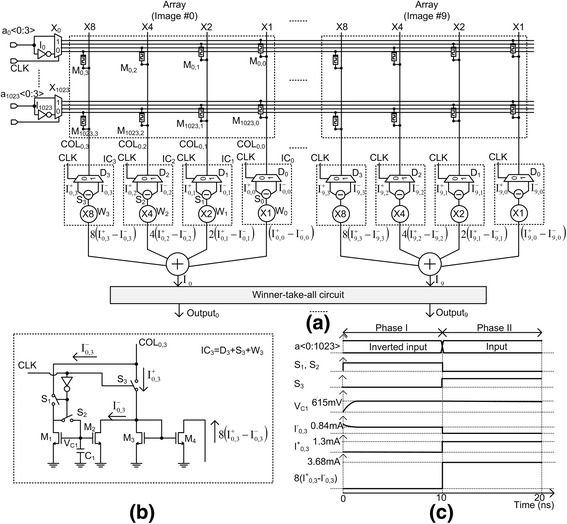
The schematic of the proposed time-shared TMC. **a** The schematic of the proposed time-shared TMC for recognizing 10 images. **b** The detailed schematic of the time-shared subtractor of IC_3_. **c** The voltage and current waveforms of the time-shared subtractor. During the phase I, I^−^ is measured and stored at C_1_. During the following phase II, I^+^-I^−^ can be calculated from recalling the I^−^ which was measured during the previous phase I

As we explained earlier, TMC is composed of two identical M^+^ arrays. These two identical arrays are applied by the input vector, *a*_0_, *a*_1_, …, *a*_*n* − 1_, and the inversion, *a* ^'^_0_, *a* ^'^_1_, …, *a* ^'^_*n* − 1_, respectively, as shown in Fig. 1a. These two arrays can be time-shared by applying *a* ^'^_0_, *a* ^'^_1_, …, *a* ^'^_*n* − 1_ and *a*_0_, *a*_1_, …, *a*_*n* − 1_, respectively, at different time, as shown in Fig. 1b. This is possible because both the input vector and its inversion are applied to the same array of M^+^ in Fig. 1b. By doing so, the time-sharing array can reduce the number of memristors by half, resulting in a great amount of area reduction. The operation of the time-shared TMC with two phases can be explained as follows. Here, for the first phase at t = k−1, we apply the inversion of input, *a* ^'^_0_, *a* ^'^_1_, …, *a* ^'^_*n* − 1_, to M^+^ array. At the following second phase at t = k, we apply the input vector, *a*_0_, *a*_1_, …, *a*_*n* − 1_, to the same M^+^ array with the previous time. By doing so, the input vector, *a*_0_, *a*_1_, …, *a*_*n* − 1_, and the inversion, *a* ^'^_0_, *a* ^'^_1_, …, *a* ^'^_*n* − 1_, can share the same M^+^ array at different time, respectively. The advantage of time-shared M^+^ array is array-area reduction. In Fig. 1b, the array area can be reduced by half, compared to two M^+^ arrays in Fig. 1a. I_0_ is the simple inverter, in Fig. 1b. Here, the multiplexer X_0_ and de-multiplexer D_0_ are controlled by the timing signal, CLK. When CLK is low, the inverted input enters the crossbar and we can obtain ${y}_j^{-}={\displaystyle \sum_{i=0}^{n-1}{a}_i^{\hbox{'}}{g}_{i, j}}$ from the de-multiplexer D_0_. When CLK is high, the input vector is applied to M^+^ and the de-multiplexer D_0_ delivers ${y}_j^{+}={\displaystyle \sum_{i=0}^{n-1}{a}_i{g}_{i, j}}$ to the time-shared subtractor S_0_ which will be shown in Fig. 2b. W_0_ is the weighting circuit in Fig. 1b. One more thing to note here is timing overhead due to the two-phase operation in Fig. 1b. The overall operation time in pattern recognition includes not only the time of crossbar array but also the time of winner-take-all circuit. Usually, because the time needed in the winner-take-all circuit is much longer than the time of crossbar operation, the overhead of two-phase operation of Fig. 1b can be ignored. Compared to negligible overhead of the two-phase operation in Fig. 1b, the array-area reduction is obviously as large as 50%.

Figure 2a shows the detailed schematic of the proposed time-shared TMC in Fig. 1b for recognizing 10 images from the image #0 to the image #9. M_0,0_, M_0,1_, M_0,2_, and M_0,3_ are memristors which correspond to the 0th pixel of the image #0. The image #0 is stored from the 0th row to 1023rd row. M_1023,0_, M_1023,1_, M_1023,2_, and M_1023,3_ are for the 1023rd pixel of the image #0. M_1023,0_, M_1023,1_, M_1023,2_, and M_1023,3_ should be weighted by ×1, ×2, ×4, and ×8, respectively, using the simple current mirror circuit, as explained in [4]. M_0,0_ is applied by a_0_<0> and the inversion a’_0_<0>, respectively, at different time, which is controlled by CLK signal. Similarly, M_0,3_ is applied by a_0_<3> and a’_0_<3>, at different time. COL_0,0_, COL_0,1_, COL_0,2_, and COL_0,3_ are for calculating the pattern-matching current of the image #0, with the weight of 1, 2, 4, and 8, respectively. In Fig. 2a, I_0_ and I_1023_ are the simple inverters, for a_0_ and a_1023_, respectively. X_0_ and X_1023_ are the multiplexers for a_0_ and a_1023_, respectively. D_3_ is the de-multiplexer for COL_0,3_. S_3_ and W_3_ are the subtractor and weighting circuit for COL_0,3_, respectively. The column current of COL_0,3_ is delivered to IC_3_ which is composed of D_3_, S_3_, and W_3_ in Fig. 2a, for COL_0,3_. The detailed schematic of IC_3_ is shown in Fig. 2b. The winner-take-all circuit can decide the best match array with the input image among 10 arrays which store 10 images, respectively.

Figure 2b shows the time-shared subtractor, IC_3_, for the column COL_0,3_ in Fig. 2a. IC_3_ is composed of D_3_, S_3_, and W_3_, as shown in Fig. 2a. The IC_3_ circuit has two phases of operation, which are the phase I and the phase II, respectively. Simply explaining, I^−^ current is measured during the phase I and I^+^–I^−^ current is calculated using the previously measured I^−^ during the phase II. If we look at Fig. 2b, the amount of ${I}_{0,3}^{-}$ is obtained from the COL_0,3_ and stored in C_1_, during the phase I, for the inverted input of *a* ^'^_0_, *a* ^'^_1_, …, *a* ^'^_*n* − 1_. At this time, S_1_ and S_2_ are on and S_3_ is off. During the following phase II, S_1_ and S_2_ are off and S_3_ is on. During this phase II, ${I}_{0,3}^{+}$ is measured from the COL_0,3_ and ${I}_{0,3}\left(={I}_{0,3}^{+}-{I}_{0,3}^{-}\right)$ is calculated by the current mirror circuit of M_1_, M_2_, and M_3_. Here, the subtraction is performed by the current of M_2_ which can recall ${I}_{0,3}^{-}$, stored at C_1_ during the previous phase I, as shown in Fig. 2b. Here, it can be noted that the de-multiplexer function can be realized by controlling three switches of S_1_, S_2_, and S_3_. The subtraction can be performed by the current mirror circuit of M_1_, M_2_, and M_3_. The weighting is realized by sizing of M_3_ and M_4_, in Fig. 2b. Similarly, ${I}_{0,2}\left(={I}_{0,2}^{+}-{I}_{0,2}^{-}\right)$, ${I}_{0,1}\left(={I}_{0,1}^{+}-{I}_{0,1}^{-}\right)$, and ${I}_{0,0}\left(={I}_{0,0}^{+}-{I}_{0,0}^{-}\right)$ are also calculated from IC_2_, IC_1_, and IC_0_, respectively, in Fig. 2a. *I*_0,3_, *I*_0,2_, *I*_0,1_, and *I*_0,0_ are added to each other and the weighted sum *I*_0_(=8*I*_0,3_ + 4*I*_0,2_ + 2*I*_0,1_ + *I*_0,0_) is delivered to the winner-take-all circuit, in Fig. 2a [3, 4]. In the winner-take-all, *I*_0_ of the image #0 is compared with the other currents of *I*_1_, …, *I*_9_ from the image #1 to the image #9.

The detailed timing diagram of the time-shared subtractor is shown in Fig. 2c. During the phase I, when S_1_ and S_2_ are on and S_3_ is off, the circuit IC_3_ in Fig. 2b measures ${I}_{0,3}^{-}$ and stores the measured amount of ${I}_{0,3}^{-}$ for the inverted input vector, at the capacitor C_1_. From Fig. 2c, V_C1_ represents the amount of current of ${I}_{0,3}^{-}$ which is converted to the capacitor’s voltage, during the phase I. During the following phase II, S_1_ and S_2_ become off and S_3_ is on. We can calculate an amount of ${I_{0,3}}^{+}-{I}_{0,3}^{-}$ by measuring *I*_0,3_^+^ and recalling ${I}_{0,3}^{-}$ which was stored at C_1_ from the previous phase I. In Fig. 2b, we used the weighting factor as large as 8, resulting in $8\times \left({I_{0,3}}^{+}-{I}_{0,3}^{-}\right)$ in Fig. 2c.

## Results and Discussion

**Fig. 3:**
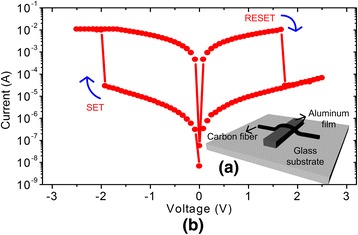
The schematic of the fabricated memristor and its voltage-current relationship. **a** The schematic of the fabricated memristor device, where carbon fiber and aluminum film crossed each other on glass substrate [8, 9]. **b** The measured current-voltage relationship that shows memristive hysteresis, in which the memristor’s voltage is swept between −2.5 and +2.5 V

The time-shared TMC proposed in this paper was verified by the fabricated 3 × 3 memristor crossbar. Figure 3a shows the fabricated single memristor which is made of carbon fiber and aluminum film [8, 9]. Here, the carbon fiber is placed on the top of thermally evaporated aluminum film like a stripe pattern. The fabrication process can be explained as follows [8, 9]. First, aluminum (Al) wire with 100 nm thickness is evaporated on a glass substrate with a 1-mm thickness. And then, a carbon fiber with 5 ~ 10-μm diameter is placed on the patterned aluminum film. The carbon fiber and aluminum film act as the top and bottom electrodes, respectively [8, 9]. The fabricated memristor demonstrated the memristive switching behavior, as shown in Fig. 3b. Here, the applied voltage is swept from −2.5 to 2.5 V and vice versa. For the positive sweep, SET-to-RESET switching can be found around 1.7 V, as shown in Fig. 3b. For the negative sweep, RESET-to-SET switching was observed around −1.8 V. The measured high-resistance state (HRS) was measured 1000 times higher than the low-resistance state (LRS) for this fabricated memristor.

**Fig. 4:**
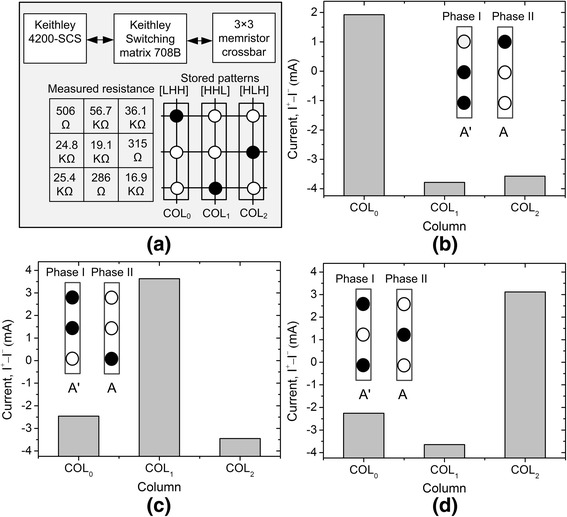
The measured results of pattern matching of the proposed time-shared TMC. **a** The measurement setup and the measured memristance values of 3 × 3 TMC. Here, the memristor crossbar were programmed to store [LHH], [HHL], and [HLH] at the *1st*, *2nd*, and *3rd columns*, respectively. **b** The measured column currents for the input vector of [LHH]. The *1st column* shows the largest amount of current among three columns. **c** The measured column currents for the input vector of [HHL]. The *2nd column* shows the largest amount of current among three columns. **d** The measured column currents for the input vector of [HLH]. The *3rd column* shows the largest amount of current among three columns

Using the fabricated memristors, 3 × 3 memristor crossbar was measured to verify the operation of the time-shared TMC proposed in this paper. The time-shared crossbar employs only one 3 × 3 array instead of two arrays as explained in Fig. 1a, b. Figure 4a shows the measurement setup for testing the time-shared TMC with 3 × 3 array. Here, we used Keithley 4200-SCS (Semiconductor Characterization System) to apply the programming and reading pulses to the memristor crossbar which has three rows and three columns. The switching matrix (Keithley 708B) is used to deliver the voltage pulses to three rows and three columns from the Source-Measure Units (SMU) of Keithley 4200. In Fig. 4a, we stored three patterns of [LHH], [HHL], and [HLH], at the three columns of crossbar, respectively. Here, “L” and “H” mean LRS and HRS, respectively.

For testing the pattern recognition of the time-shared TMC, we applied three different input vectors to the crossbar, which are [LHH], [HHL], and [HLH], respectively. Figure 4b shows the measured currents of three columns when we apply the input vector [LHH] and its inversion [HLL] to the time-shared crossbar, respectively. The measurement shows that the first column’s current is the largest among the three columns. Thus, the following winner-take-all circuit can choose the first column as a winner. When we apply the input vector [HHL] and the inversion [LLH], respectively, at different time, the time-shared subtractor measured the *I* ^+^ − *I* ^−^ values for three columns. Comparing the three currents, the measurement shows the second column has the largest current, as shown in Fig. 4c. Similarly, the third column was measured to have the largest current among three columns, for the input vector [HLH] and its inversion [LHL], as indicated in Fig. 4d.

**Fig. 5:**
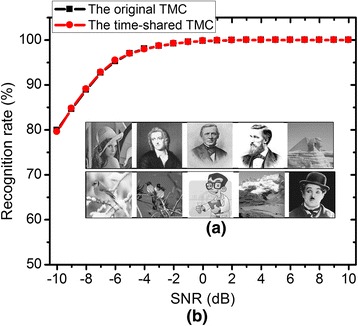
The simulation results of the proposed time-shared TMC for image recognition application. **a** The tested images shown in the *inset*. **b** The comparison between the original TMC and the time-shared TMC for recognizing 10 images. Here, the signal-to-noise ratio is varied from −10 to +10 dB

For verifying the image recognition of the time-shared TMC, we designed 1024 × 40 memristor crossbar with the CMOS time-shared subtractor and the CMOS winner-take-all circuit, as shown in Fig. 2a, b. The memristor crossbar and CMOS circuits were simulated together by the circuit simulator (CADENCE/SPECTRE) [10]. Here, the memristive behavior was modeled by Verilog-A in the circuit simulator [11]. The time-shared subtractor and winner-take-all circuit were designed by the commercial CMOS technology which was obtained from SAMSUNG 0.13-μm process. The tested images with 32 × 32 pixels are shown in Fig. 5a. Figure 5b compares the recognition rate between the original TMC and the proposed time-shared TMC for the recognition of 10 images. Here, the Gaussian noise is added to the tested images, in which the signal-to-noise ratio (SNR) varies from −10 to +10 dB for each image. From Fig. 5b, we can know that the proposed time-shared TMC and the original TMC show the same recognition rate for the 10 tested images. This simulation verifies that the proposed time-shared TMC has the same performance in pattern recognition with the original TMC.

## Conclusions

In this paper, we proposed the time-shared TMC for pattern-recognition applications. By sharing two memristor arrays at different time, the number of memristor arrays can be reduced by half, saving the crossbar’s area by about half. To implement the time-shared TMC, we designed and verified the CMOS time-shared subtractor by the circuit simulation. The operation of the time-shared TMC was experimentally verified using the fabricated 3 × 3 memristor array which was made of aluminum film and carbon fiber. Here, we programmed the array to store three different patterns. By applying three different input vectors to the time-shared TMC, we could verify that the input vectors were recognized well by the proposed circuits. Moreover, the proposed time-shared TMC was tested for the recognition of more complicated gray-scale images. Here, 10 gray-scale images with 32 × 32 pixels were tested and verified to be recognized well by the proposed time-shared TMC, even though the noise level was varied from −10 to +10 dB.
